# Mdm2/p53 levels in bone marrow mesenchymal stromal cells Is essential for maintaining the hematopoietic niche in response to DNA damage

**DOI:** 10.21203/rs.3.rs-2544760/v1

**Published:** 2023-03-02

**Authors:** Rasoul Pourebrahim, Rafael Heinz Montoya, Zoe Alaniz, Lauren Ostermann, Patrick P. Lin, Bin Liu, Edward Ayoub, Jared K. Burks, Michael Andreeff

**Affiliations:** MD Anderson Cancer Center; MD Anderson Cancer Center; MD Anderson Cancer Center; MD Andreson Cancer Center; MD Anderson Cancer Center; Epigenetic and Molecular Carcinogenesis; Leukemia; Leukemia; The University of Texas MD Anderson Cancer Center

**Keywords:** Mdm2, p53, MSCS, mouse model, osteogenesis, glycolysis, bone marrow fibrosis

## Abstract

Mesenchymal stromal cells (MSCs) are a key component of the bone marrow (BM) niche, providing essential support required for maintenance of hematopoietic stem cells. To advance our understanding of physiological functions of p53 and Mdm2 in BM-MSCs, we developed traceable conditional mouse models targeting *Mdm2* and/or *Trp53 in vivo*. We demonstrate that Mdm2 is essential for the emergence, maintenance and hematopoietic support of BM-MSCs. *Mdm2* haploinsufficiency in BM-MSCs resulted in genotoxic stress-associated thrombocytopenia, suggesting a functional role for Mdm2 in hematopoiesis. In a syngeneic mouse model of acute myeloid leukemia (AML), *Trp53* deletion in BM-MSCs improved survival, and protected BM against hematopoietic toxicity from a murine Mdm2i, DS-5272. The transcriptional changes were associated with dysregulation of glycolysis, gluconeogenesis, and Hif-1α in BM-MSCs. Our results reveal a physiologic function of Mdm2 in BM-MSC, identify a previously unknown role of p53 pathway in BM-MSC–mediated support in AML and expand our understanding of the mechanism of hematopoietic toxicity of MDM2is.

## Introduction

Wild-type p53 functions are frequently suppressed by murine double minute 2 (MDM2) protein, an E3 ubiquitin ligase that targets p53 for proteasome degradation ^1, 2^. Genetic studies by global or tissue-specific *Mdm2* deletion have established the primary function of Mdm2 in regulating p53 and have shown that the lethal phenotype of *Mdm2* loss was attributed to increased p53 activity ^3^. We previously showed that Nutlin-3a-mediated p53 activation in BM-MSCs downregulates *Cxcl12* expression via HIF-1α pathway ^4^. Cxcl12, also called stromal cell-derived factor 1 or SDF-1, is secreted by perivascular MSCs and regulates homing, proliferation, and differentiation of hematopoietic stem cells ^5, 6, 7, 8^. In addition, while the restoration of p53 activity by MDM2 inhibitors (MDM2i) represents a promising approach in cancer therapy, little information regarding the effect of these drugs on the BM microenvironment is available.

Homozygous deletion of *Mdm2* in mature osteoblasts using *Col3.6-Cre* resulted in skeletal defects, reduced bone formation and lethality before birth ^9^. p53-null mice displayed a significant increase in the osteogenic differentiation potential of MSCs, mediated in part by upregulation of osteoprotegerin ^10^. *Mdm2* haploinsufficient mice typically were not distinguishable from wild-type mice except for their hematopoietic failure following ionizing radiation (IR), although it could not be determined whether BM failure after IR was caused directly by cytotoxic effects on hematopoietic stem cells or indirectly through alterations in MSCs-mediated hematopoietic support ^11, 12^.

In addition to the role of p53 in cell cycle arrest and apoptosis, a growing body of evidence suggests that activation of p53 in the tumor microenvironment following MDM2i treatment could result in stromal senescence and an immunosuppressive microenvironment ^13, 14, 15^. MSCs derived from patients with acute myeloid leukemia (AML), but not normal MSCs, highly express p53, suggesting that the p53 pathway is active in the leukemia microenvironment ^16^, although the extent to which p53 activity in MSCs is relevant to AML progression is poorly understood.

Here we investigated whether Mdm2/p53 levels in MSCs contribute to MSCs survival, differentiation, and hematopoietic support *in vivo*. Several *in vivo* systems were used to identify Mdm2 functions in MSCs and their support in hematopoietic maintenance. We demonstrated that the hematopoietic toxicity of MDM2i therapy is in part due to p53 activation in MSCs and that deletion of *Trp53* in MSCs can prevent such deleterious side effects and improve response to therapy. These observations illustrate a previously unappreciated function of the Mdm2/p53 pathway in differentiation of MSCs and their role in hematopoietic support.

## Results

### Mdm2 levels are essential for the survival of MSCs

We first sought to generate a BM-specific MSC reporter mouse to mark MSCs and perivascular cells *in vivo*. Because of its previously characterized expression in other settings, we used the *Osx-Cre* allele ^17, 18, 19^ combined with *mTmG* allele ^20^ to generate double-transgenic *Osx-Cre;mTmG* reporter mice. The *mTmG* allele expresses membrane-localized red fluorescence globally in the absence of *Cre* recombinase and green fluorescence specifically in *Cre* recombinase-expressing cells. Consistent with previous reports ^17, 18^, GFP-expressing cells were exclusively present in MSCs that give rise to BM stromal cells and eventually differentiate to osteoblasts and adipocytes (Supplementary Figure S1A). In adult BM, GFP marked the osteoblast lineages including trabecular, endosteal, and periosteal cells. The Lepr+ MSCs and perivascular cells within the BM were green fluorescent protein positive (GFP+) (Supplementary Figure S1B). Flow cytometric analysis of markers for MSCs in GFP+ cells derived from BM showed that the GFP+ cells were partially positive for CD73, CD44, and CD90, suggesting that the *Osx-Cre;mTmG* reporter accurately marked the population of MSCs (Supplementary Figure S1C). Thus, the population of GFP+ cells marked by *Osx-Cre* represent the major characteristics of MSCs in postnatal mice.

**Figure 1:**
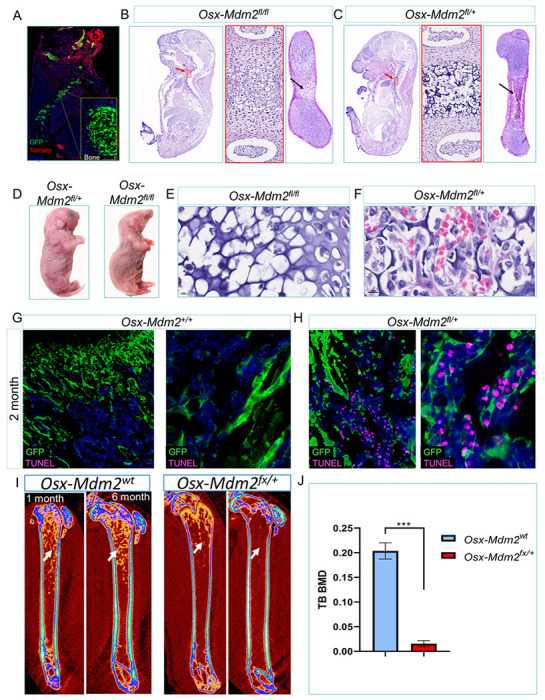
Mdm2 levels are essential for the survival of MSCs (A) Direct fluorescence microscopy image of sagittal body section of an *Osx-cre;mTmG*mouse showing the exclusive expression of GFP in neonatal bones at E18.5. Yellow box marks the higher magnification of the indicated bone. Extraskeletal tissues were GFP negative. (B and C) Microscopic views of sagittal sections of the indicated embryos at E18.5. High magnification of active osteogenesis areas is marked by the red arrow and the red box. The black arrows show the osteogenesis in the bones. (D) Representative image of the newborns with indicated genotypes. (E and F) Microscopic view of bone metaphysis in indicated genotypes. (G and H) Immunofluorescence images of TUNEL staining in indicated mice. The high magnification view is shown on right. (I) Micro-CT image of bones derived from the indicated mice at the indicated ages showing the distribution of trabecular bones in each mouse. The white arrow points to the trabecular bone density. (J) Graph bar showing the trabecular bone density in indicated mice (n = 3, mean ± SD); ***p < 0. 001, Student’s *t* test.

Next, we evaluated the role of Mdm2 in MSCs by conditional deletion of *Mdm2* using *Osx-Cre* as the driver. We interbred progeny of *Osx-Cre;mTmG* mice with the *Mdm2*^*fl*^ allele ^21^ so that we were able to delete *Mdm2* specifically in MSCs and trace them with GFP (Figure 1A). Then, we evaluated the bone and hematopoietic phenotype of littermate embryos derived from crossing of the *Mdm2*^*fl/fl*^ mice with the *Osx-Mdm2*^*fl/+*^ mice. Mice with homozygous deletion of *Mdm2, Osx-Cre;Mdm2*^*fl/fl*^, died shortly after birth due to skeletal malformation and compromised breathing (Figure 1D). Developing bones in the *Osx-Mdm2*^*fl/fl*^ mice displayed enlarged chondrocytes without ossification and trabecular bone formation (Figure 1B). However, the *Osx-Mdm2*^*fI/+*^ mice did not show any abnormalities in trabecular bone formation and the structure of the growth plate (Figure 1C). Importantly, hematopoiesis was eliminated in the metaphysis of developing bones in *Osx-Mdm2*^*fl/fl*^ mice, suggesting that Mdm2 is essential for hematopoietic support by MSCs (Figure 1E). The hematopoietic cells were distributed throughout the metaphysis of developing bones in *Osx-Mdm2*^*fl/+*^ mice (Figure 1F).

Since mice with homozygous deletion of *Mdm2* in MSCs were not viable, we focused on *Osx-Mdm2*^*fl/+*^ mice and further characterized their bone development. We used terminal deoxynucleotidyl transferase-mediated dUTP nick-end labeling (TUNEL) staining to estimate DNA strand breaks as a marker of cell apoptosis in bone cells. Many MSCs, but not mature osteocytes, in Osx-*Mdm2*^*fl/+*^ femurs were TUNEL positive, whereas few TUNEL positive cells were seen in the femurs of Osx-*Mdm2*^*+/+*^ mice (Figures 1G and 1H). To evaluate the osteoblast differentiation potential of MSCs in *Osx-Mdm2*^*fl/+*^ mice, we performed bone densitometry. The trabecular bone volume in Osx-*Mdm2*^*fl/+*^ mice was significantly decreased significantly more than that of similarly-aged Osx-*Mdm2*^*wt*^ mice (Figure 1I and 1J), suggesting that osteoblast differentiation of MSCs was also attenuated in *Osx-Mdm2*^*fl/+*^ mice. Thus, genetic depletion of Mdm2 in MSCs was associated with increased MSCS apoptosis levels and osteopenia in adult mice.

### *Mdm2* haploinsufficiency in MSCs promotes thrombocytopenia after IR-induced cellular stress

**Figure 2:**
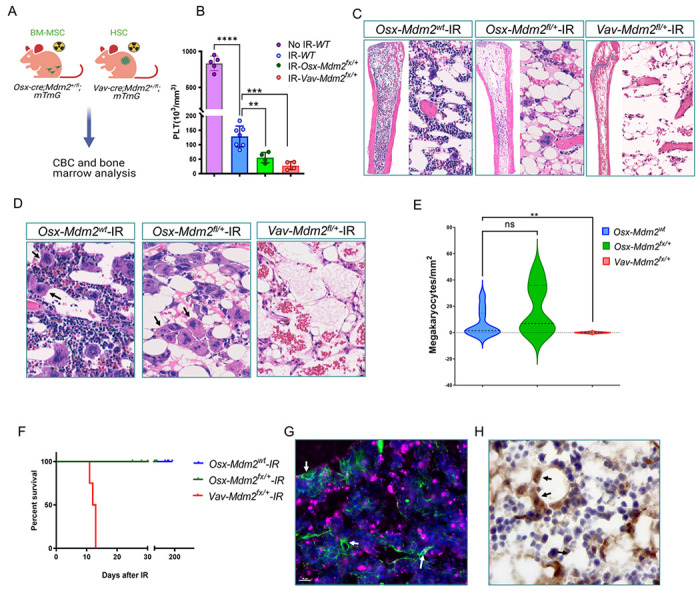
Mdm2 haploinsufficiency in MSCs promotes thrombocytopenia after IR induced cellular stress (A) Schematic view of experiment design. (B) Bar graph showing platelet counts in the indicated mice 10 days after irradiation. Data points represent the platelet count in each mouse, (mean ± SD); **** p < 0.0001, *** p < 0.001, ** p < 0.01, Student’s *t* test. (C) Representative microscopic view of hematoxylin and eosin (H&E) staining of BM sections isolated from the indicated mice 2 weeks after IR. (D) High magnification microscopic view of H&E staining of BM sections in indicated mice after IR. The black arrows point to megakaryocytes. Kaplan-Meier plot of the indicated mice after irradiation. (E) Quantification of the megakaryocytes from D (control n = 10; *Osx-Mdm2*^*fl/+*^ n = 6; *Vav-Mdm2*^*fl/+*^ n = 6). **P < 0.01. (G) TUNEL staining of a BM sample derived from *Osx-Cre;mTmG* mouse after IR. The white arrows point to MSCs (GFP+). (H) Immunostaining of p53 in *Osx-Mdm2*^*fl/+*^ mouse after irradiation.

We examined whether the Mdm2 level in MSCs was important in hematopoiesis. The population of hematopoietic stem cells defined as CD150^+^Lin^−^/c-Kit^+^/Sca-1^+^ was not significantly altered in the BM of Osx-*Mdm2*^*fl/+*^ mice compared with control *Osx-Mdm2*^*+/+*^ mice, suggesting that the hematopoietic support of MSCs was not functionally compromised in Osx-*Mdm2*^*fl/+*^ mice (Supplementary Figure S2A). To determine whether the Mdm2 level in MSCs was important in hematopoietic recovery after cellular damage by irradiation, the *Osx-Mdm2*^*fl/+*^ and control mice were irradiated (6 Gy), and peripheral blood was analyzed by cell counter. We included a cohort of hematopoietic-specific *Mdm2* haploinsufficient (*Vav-Cre;Mdm2*^*fl/+*^) mice, known to be IR-sensitive, as positive controls (Figure 2A). Analysis of the peripheral blood one week after IR showed a significant decrease in peripheral platelet counts after IR in all mice (Figure 2B). There were no significant differences between *Osx-Mdm2*^*fl/+*^ (*Mdm2* haploinsufficient MSCs) and *Vav-Cre;Mdm2*^*fl/+*^ (*Mdm2* haploinsufficient hematopoietic stem cells) in response to IR, as both groups developed severe thrombocytopenia. However, platelet counts were significantly lower in *Osx-Mdm2*^*fl/+*^ mice than in the *Mdm2*^*wt*^ controls (p < 0.001), suggesting that *Mdm2* haploinsufficiency in MSCs contributed to platelet production by megakaryocytes during the recovery phase. Analysis of BM sections derived from irradiated mice revealed a striking pancytopenia in *Osx-Mdm2*^*fl/+*^ as well as *Vav-Cre;Mdm2*^*fl/+*^ mice compared with controls (Figure 2C). The megakaryocytes were absent in *Vav-Cre;Mdm2*^*fl/+*^ mice (Figure 2D). However, the number of megakaryocytes were comparable between *Osx-Mdm2*^*fl/+*^ and the controls, suggesting that the thrombocytopenia after irradiation was due to the functional impairment of megakaryocytes in platelet production rather than their depletion (Figures 2D and 2E). In contrast to *Osx-Mdm2*^*fl/+*^mice, which survived after irradiation, all *Vav-Cre;Mdm2*^*fl/+*^ mice died around 2 weeks after IR, likely due to eradication of the hematopoietic stem cells (Figure 2F). TUNEL staining of BM samples derived from *Osx-Mdm2*^*fl/+*^ mice after IR showed apoptosis of hematopoietic cells, whereas the MSCs were completely devoid of TUNEL positivity, indicating that *Mdm2* haploinsufficient MSCs survived the DNA damage following irradiation, and that the thrombocytopenia observed in these mice was not due to loss of MSCs (Figure 2G). In addition, immunostaining showed strong p53 accumulation in MSCs of *Osx-Mdm2*^*fl/+*^ mice (Figure 2H), suggesting that *Mdm2* haploinsufficiency results in accumulation of p53 after IR stress. Thus, inhibition of Mdm2 in MSCs contributes to thrombocytopenia after IR.

### Deletion of *Trp53* in MSCs prevents the myelosuppressive side effects of MDM2i

**Figure 3:**
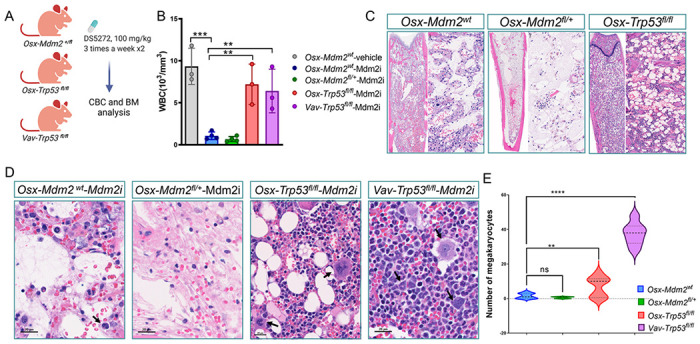
Deletion of *Trp53* in MSCs prevents the hematopoietic toxicity of MDM2i. (A) Schematic view of the experiment design. (B) Bar graph showing white blood cell (WBC) counts in indicated mice after the last dose of treatment. Data points represent the WBC count in each mouse, (mean ± SD); ***P < 0.0001, **P < 0.01, Student’s *t* test. (C) Representative microscopic view of H&E staining of BM sections isolated from indicated mice after the last dose of treatment. The right panels display higher magnification. High magnification view of H&E staining of BM sections in red box. (D) Quantification of the megakaryocytes from C (controls n = 3; *Osx-Mdm2*^*fl/+*^ n = 4; *Osx-Trp53^fl/fl^* n = 3; V*av-Trp55^fl/fl^* n = 3); ****P < 0.0001, **P < 0.01, Student’s *t* test.

We next examined whether the myelosuppressive effects of MDM2i are due to p53 levels in MSCs. Mice with specific deletion of *Trp53* (*Osx-Trp53*^*fl/fl*^)or *Mdm2* (*Osx-Mdm2*^*fl/+*^) in MSCs and control mice with hematopoietic-specific deletion of *Trp53* (*Vav-Cre;Trp53^fl/fl^* were treated with DS5272, an orally active murine Mdm2i ^22^, and peripheral blood and BM samples were analyzed after the last dose of treatment (Figure 3A). Mdm2i treatment resulted in leukopenia in peripheral blood of mice in a p53-dependent manner (Figure 3B). Heterozygous deletion of Mdm2 in MSCs exacerbated the myelosuppressive effect of MDM2i, however, deletion of *Trp53* in MSCs (*Osx-Cre;Trp53*^*fl/fl*^) completely reversed the cytopenia associated with Mdm2i treatment (Figure 3B), In fact, p53 deletion in MSCs was equally efficient as deletion of *Trp53* in hematopoietic cells (*Vav-cre-Trp53*^*fl/fl*^), suggesting that increased p53 levels in MSCs contributed to hematopoietic toxicities of MDM2i therapy. Analysis of BM samples revealed moderate cytopenia in Mdm2^wt^ mice and striking cytopenia in *Osx-Mdm2*^*fl/+*^ mice after MDM2i treatment (Figure 3C). Importantly, the BM of mice with deletion of *Trp53* in MSCs (*Osx-Cre;Trp53*^*fl/fl*^) displayed areas of active hematopoiesis and high cellularity (Figure 3C). The megakaryocytes were completely depleted in *Osx-Mdm2*^*fl/+*^ mice, whereas *Osx-cre;Trp53*^*fl/fl*^ mice displayed a significantly higher number of megakaryocytes in the BM (p < 0.01, n = 3). Collectively, these data demonstrate that p53 levels in MSCs are functionally important in hematopoietic failure after MDM2i therapy.

### p53 in MSCs contributes to response to murine MDM2i

**Figure 4:**
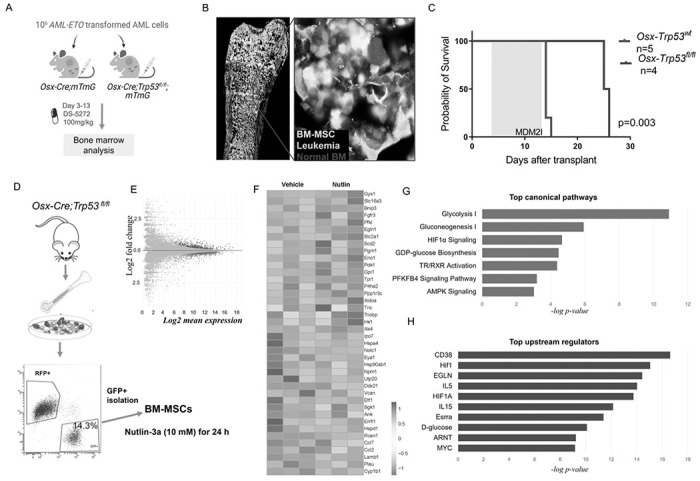
p53 in MSCs contributes to response to murine Mdm2 inhibitor (A) Schematic view of the experimental design. (B) Direct fluorescence microscopy image of a longitudinal section of femur derived from a syngeneic *Osx-Cre;Trp53*^*fl/fl*^ AML mouse showing MSCs (green), leukemia cells (turquoise), normal hematopoietic cells (red) and DAPI (blue). The red box marks the region of interest shown in the right panel. (C) Kaplan-Meier survival curves of recipient mice reconstituted with p53-null AML1/ETO9a transformed leukemia cells. The treatment period is marked by the blue box. (D) Diagram showing the origin and isolation process of MSCs treated with MDM2 inhibitor or vehicle. (E) Mean-average plot of Log_2_ mean expression versus Log2 fold change between treated and vehicle-treated MSCs. Red dots indicate genes with significantly higher expression in treated MSCs (Log_2_FC > 1 and *P* < 0.05). Blue dots indicate genes with significantly lower expression in treated MSCs (Log_2_FC < −1 and *P* < 0.05). (F) Heat map representing the transcriptome alteration in the indicated groups. log_2_ expression values, n = 3. p < 0.01. (G) Top predicted annotations for pathways and functions ranked by Z-score by Ingenuity Pathway Analysis (IPA) for the top 20 differentially expressed genes. (H) IPA upstream regulator analysis on differentially expressed genes shown in F.

Previously, we reported that MSCs derived from AML patients display significantly higher p53 protein levels ^16^. To determine whether p53 levels in MSCs contribute to the response to MDM2i, we established a traceable syngeneic leukemia model in *Osx-Cre;mTmG* and *Osx-Cre;mTmG;Trp53*^*fl/fl*^ mice and analyzed their survival. Mice were transplanted with leukemia cells originally derived from p53-null mice and transformed by lentivirus-mediated delivery of an oncogenic *AML-ETO* fusion gene ^23^ as well as a fluorescent transgene to express turquoise fluorescence (Figure 4A). We validated the *Cre* activity and engraftment of leukemia cells by fluorescent microscopic analysis of BM isolated from syngeneic AML mice 10 days after transplant (Figure 4B). Mice were treated with vehicle or DS-5272 starting on day 3 after transplant for 10 days. Deletion of p53 in MSCs significantly prolonged mice’s survival beyond discontinuation of therapy (p < 0.003), suggesting that survival of AML cells in response to DS-5272 might depend on p53 levels in MSCs (Figure 4C). Of note, Mdm2 inhibition with nutlin-3a has been shown to disrupt p73-MDM2 interaction in p53-null cells ^24^. To assess the early molecular pathways in p53-null MSCs after Mdm2 inhibition, we isolated GFP+ cells from *Osx-Cre;Trp53*^*fl/fl*^ mice (p53 null) and determined the gene expression profiles of vehicle- or nutlin-treated cells after 24 hours (Figure 4D). RNA-Seq and subsequent Ingenuity Pathway Analysis of p53null MSCs revealed upregulation of genes involved in glycolysis as well as Hif-1α signaling (Figures 4E–4G). The top seven pathways identified by the upregulated differentially expressed genes are illustrated in Figure 4G. Mdm2 inhibition induced *Slc2a1* (*Glut1), Pdk1*, and *Fgfr3* genes, known to promote osteoblast differentiation ^25, 26, 27^. *Scd2* (stearoyl-CoA desaturase 2) plays a role in the regulation of energy metabolism and lipid synthesis and was significantly upregulated by Mdm2 inhibition. The top upstream activated regulator was CD38, a key cellular metabolic driver of aging ^28^ (Figure 4H). In addition to CD38, Hif-1, Egln, IL5, and IL15 were enriched as upstream regulators of genes induced in p53-null MSCs treated with Mdm2i. These expression changes suggest that Mdm2 inhibition might promote osteoblast differentiation of p53-null MSCs partly through metabolic pathways.

### Heterozygous deletion of *Mdm2* in *Osx-Trp53P^fl/fl^* mice results in osteosclerosis and myelofibrosis

**Figure 5:**
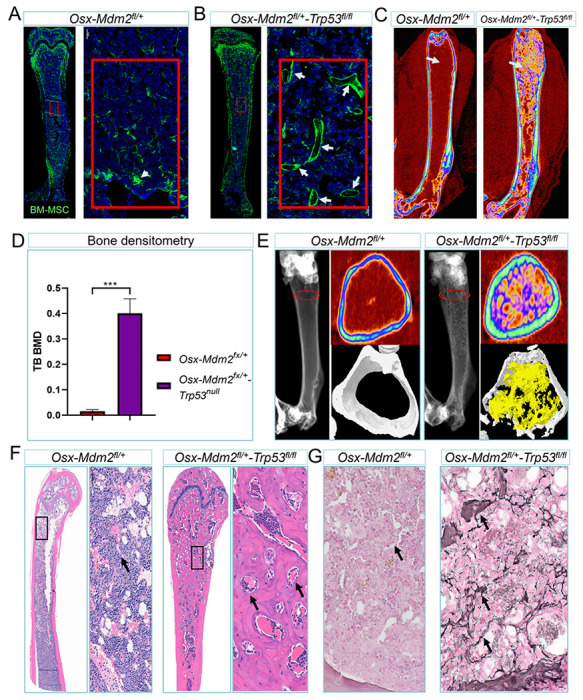
Deletion of p53 in *Osx-Mdm2*^*fl/+*^ mice results in osteosclerosis (A) Direct fluorescence image of a bone section derived from an 8-week-old *Osx-Mdm2*^*fl/+*^*;mTmG* mouse showing the population of MSCs as GFP+ as well as trabecular bone formation (white arrow). The red box area is magnified in the adjacent panel. (B) Direct fluorescence image of a bone section derived from a 8 weeks-old *Osx-Cre;Trp*^*fl/fl*^*;Mdm2*^*fl/+*^*;mTmG* mouse showing the population of MSCs as well as trabecular bone formation (white arrows). The red box area is magnified in the adjacent panel. (C) Micro-CT scan of bones in the indicated mice at 6 months of age showing the trabecular bone formation inside the BM cavity. (D) Bar graph showing the quantification of trabecular bone density in the indicated mice (n = 3; *** p < 0.001, Student’s *t* test). (E) Histomorphometry of bones isolated from indicated mice showing the density of trabecular (yellow) and cortical bone (white) at the level marked by red dash line. (F) Microscopic view of H&E staining of bones derived from the indicated mice. The black box is magnified in the adjacent image. The black arrows mark the hematopoietic cells trapped inside trabecular bone. (G) Representative image of reticulin staining of bones derived from the indicated mice. The black arrows mark the reticulin positive cells.

To determine the direct effect of Mdm2 inhibition in *Osx-Trp53*^*fl/fl*^ mice, we interbred progeny from *Osx-Mdm2*^*+/fl*^ and *Trp53*^*fl*^ mice with *mTmG* reporter mice to generate *Osx-Cre;Mdm2*^*+/fl*^;*Trp53*^*fl/fl*^*;mTmG* mice (referred to hereafter as *Osx-Mdm2*^*+/fl*^*;Trp53*^*fl/fl*^). This enabled us to image the population of MSCs (GFP+) irrespective of their cell surface markers that change upon their differentiation. As expected, homozygous deletion of *Trp53* reversed the prenatal lethal phenotype of *Osx-Mdm2*^*fl/fl*^ mice, and pups were born at Mendelian ratios without an obvious bone phenotype (data not shown). Unlike *Osx-Mdm2*^*+/fl*^ mice, in which we had observed less trabecular bone volume, *Osx-Mdm2*^*+/fl*^*;Trp53*^*fl/fl*^ mice displayed trabecular bone formation mainly due to trabecular ossifications, resulting in a sponge-like network of trabecular bone (Figures 5A–5C). Bone densitometric analysis confirmed a sclerotic trabecular bone phenotype in the *Osx-Mdm2*^*+/fl*^*;Trp53*^*fl/fl*^ mice, accompanied by significant increase in trabecular bone volume (Figures 5C–5D). Bone histomorphometric further confirmed the ossification of trabecular bones in *Osx-Cre;Mdm2*^*+/fl*^*;Trp53*^*fl/fl*^ mice (Figure 5E). *Osx-Mdm2*^*+/fl*^*;Trp53*^*fl/fl*^ mice became moribund as they aged and died of BM failure. Histopathologic analysis of the BM in *Osx-Mdm2*^*+/fl*^*;Trp53*^*fl/fl*^ mice revealed massive endochondral bone formation that reduced the effective marrow space by approximately 80%. The architecture of the growth plate displayed typical chondrocytes with regular proliferating and hypertrophic zones. However, osseous trabeculae with new bone formation were present throughout the epiphysis, metaphysis, and diaphysis of the long bones as well as vertebrae (Figure 5F). The diaphyseal cortical bone diameter was increased and coalesced with the subjacent trabecular bone. Histologic examination by reticulin staining showed widespread reticulin positivity in the BM reminiscent of myelofibrosis (Figure 5G). Of note, the trabecular bone volume in mice with deletion of p53 in MSCs, *Osx-cre;Trp53*^*fl/fl*^, was comparable with that of p53 wild-type mice, suggesting that the observed phenotype in *Osx-Mdm2*^*+/fl*^*;Trp53*^*fl/fl*^ mice was due to decreased levels of Mdm2 (Supplementary Figures S2B and S2C).

Next, we sought to determine whether the sclerotic BM was derived from MSCs. Since the sclerotic bones were densely mineralized, we decided to perform immunohistochemistry for GFP in the BM sections isolated from *Osx-Cre;mTmG;Mdm2^+/ff^;Trp53^fl/fl^* mice. As shown in Supplementary Figure S2D, the sclerotic BM was GFP positive, suggesting that the sclerotic trabecular bones were derived from MSCs. Collectively, these data demonstrate that genetic depletion of *Mdm2* in MSCs lacking p53 promotes osteoblast differentiation leading to lethal osteosclerosis.

## Discussion

Several new selective MDM2is have been developed and advanced into early phase clinical trials in different cancers, with promising results ^29, 30, 31^. However, dose-limiting hematopoietic toxicities such as thrombocytopenia often compromise treatment efficacy, and therefore ineffective treatment is common. We investigated the effects of Mdm2 deficiency on MSCs and explored the role of p53 in MSCs in drug-related cytopenia. First, by using traceable conditional animal models, we demonstrated that heterozygous deletion of *Mdm2* resulted in osteopenia. Second, we presented genetic evidence that Mdm2 levels are crucial in the differentiation of MSCs, particularly in the absence of p53. Third, we demonstrated that p53 levels in MSCs are important in Mdm2i-associated cytopenia. Together, these data identify an important role for Mdm2/p53 in the homeostasis of MSCs and their hematopoietic support.

We developed a genetic model enabling the identification and imaging of multipotent stromal cells *in vivo*. The population of MSCs marked by *Osx-Cre* were CD45 negative, as previously described ^18^. Other studies have reported MSCs in reporter mice with use of Gli1-cre mice ^32^, transgenic CD73-EGFP BAC mice ^33^, and nestin-Cre mice ^34^. Gli1-cre and CD73-EGFP reporter mice enabled labeling of presinusoidal endothelial cells, whereas expression of the reporter was minimal in LepR+ cells.

A previous report of mice bearing a different *Mdm2*-floxed allele driven by *Col3.6-Cre* revealed multiple skeletal defects and reduced bone length; however, osteoblasts deleted for *Mdm2* did not undergo apoptosis even though they exhibited elevated p53 activity ^9^. Our data indicate that homozygous deletion of Mdm2 completely blocks osteogenesis and that Mdm2 is essential for osteoblast differentiation.

Another intriguing phenotype in Osx-*Mdm2*^*fl/+*^ mice is the fitness selection of emerging MSCs in early development. Osx-*Mdm2*^*fl/+*^ mice displayed apoptosis of MSCs in early developing bones at E18.5; however, the population of MSCs was compensated and was able to reconstitute the developing bones. These data suggest that the Mdm2/p53 pathway maybe involved in the cell competition process during early bone development, as previously reported in other tissues ^35^.

Mice with loss of one copy of *Mdm2* in their MSCs displayed osteopenia mainly in their trabecular bone compartment, which is known to be derived from MSCs ^36^. Deletion of *Trp53* reversed the phenotype, and robust osteogenesis occurred with perturbation of Mdm2 and p53. The observed hyperostosis phenotype was not present in *Osx-Cre;Trp53*^*fl/fl*^ mice, indicating a role of Mdm2 in the differentiation of MSCs to osteoblasts and fibroblasts. In addition, our data show that myelofibrosis observed in *Osx-Mdm2*^*fl/+*^*;Trp53*^*fl/fl*^ mice originated from MSCs. Recently, Leptin receptor–expressing MSCs were identified as the source of myofibroblasts in primary myelofibrosis ^37^.

We present genetic evidence identifying a previously unappreciated role of Mdm2 in the differentiation of MSCs lacking p53. *Mdm2* is commonly thought of as an essential gene only when p53 is competent and thus is regarded erroneously to be unnecessary when p53 is absent. Considering that *Mdm2* levels are regulated by p53, our data support the hypothesis that downregulation of Mdm2 upon p53 deletion may be important for cellular differentiation. Mechanistically, we demonstrate that the glycolysis and Hif-1α pathways are upregulated in p53-null MSCs upon MDM2i treatment. Mdm2 inhibition has been reported to actively downregulate Hif-1α through p53 activation ^38^. We previously showed that Hif-1α plays a role in the nutlin-mediated downregulation of Cxcl12 ^4^. In addition, stabilization of Hif-1α in Osx-positive cells in postnatal mice was shown to stimulate trabecular bone formation and glycolysis by upregulating pyruvate dehydrogenase kinase 1 (PDK1), a key glycolytic enzyme ^39^. We previously reported that PDK1 is significantly upregulated in MSCs derived from patients with AML ^16^. In addition, *Glut1* expression is increased in osteoblasts to switch their metabolic pathway to glycolysis during differentiation ^40^. We observed that inhibition of Mdm2 in cells lacking p53 can induce several glycolytic enzyme-coding genes other than *Slc2a1* (*Glut1*), including *Hk1, PfkI*, and *Slc16a3*, to convert the metabolism to glycolysis. The p53-independent role of Mdm2 in cellular metabolism and its role in the regulation of cell differentiation remain to be explored in future studies.

The hematopoietic toxicities associated with MDM2is such as nutlin-3 have been attributed to the involvement of MDM2 in hematopoiesis ^41^. We observed that the level of Mdm2 in MSCs is important in hematopoietic failure due to MDM2i therapy. Importantly, deletion of p53 in MSCs completely blocked the hematopoietic failure upon MDM2i treatment, suggesting that targeting p53 activity in MSCs can be therapeutically beneficial in the context of MDM2i-associated hematopoietic toxicity. Thus, MDM2i can affect the BM microenvironment to induce various cellular responses. It remains to be explored whether the increased p53 levels in MSCs upon challenge with genotoxic drugs may contribute to hematopoietic failure after therapy in the same way.

In summary, our study sheds light on the importance of the Mdm2/p53 pathway in the differentiation of MSCs and BM hemostasis. The balance between Mdm2 and p53 is crucial in maintaining MSCs as well as to the survival of hematopoietic cells, which are dependent on MSCs. Thus, the role of the Mdm2/p53 pathway in homeostasis of the BM microenvironment could have important therapeutic ramifications.

## Material, Subjects, And Methods

### Mice

Mdm2-floxed mice (*Mdm2*^*tm2.1Glo*^/J), mTmG mice (B6.129(Cg)-*Gt(ROSA)26Sor^tm4(ACTB-tdTomato,-EGFP)Luo^*/J), Trp53-floxed mice (B6.129P2-*Trp53*^*tm1Brn*^/J ), Osx-Cre mice (B6.Cg-Tg(Sp7-tTA,tetO-EGFP/Cre)1Amc/J), and Vav-Cre mice (B6.Cg*Commd10^Tg(Vav1-icre)A2Kio^*/J ) were purchased from The Jackson Laboratories. Animals were housed in the MD Anderson Cancer Center animal facility, and all procedures using animals were approved by the Institutional Animal Care and Use Committee. For MDM2i treatment, mice were treated by oral gavage with DS5272 (Daiichi Sankyo, 100 mg/kg, three times/week) for two weeks and euthanized by asphyxiation with CO_2_ for analysis 24 hours after the last dose.

### Flow cytometry

Fresh BM single-cell suspensions were prepared as described previously ^42^. Marrow and bone fractions were pooled and digested for 25 minutes at 37°C with 1 mg/mL STEMxyme1 (Worthington, LS004106) and 1 mg/mL Dispase II (Thermo Fisher Scientific, 17105041), in Media 199 with 2% fetal bovine serum and agitation. After digestion, bone fractions were filtered through 40-μm strainers (Fisher, 08-771-1). Red blood cells (RBCs) were lysed in 1× red blood cell lysis buffer (BD Biosciences, 555899) at room temperature for 15 minutes with slight agitation, washed twice in phosphate-buffered saline (PBS, Invitrogen, catalogue number FB002), and stained with following antibodies: CD73-AF647 (BD 561543), CD44-APC-Cy7 (BD 560568), CD90-PB (BioLegend 140305), and ghost dye 540 (Tonbo Biosciences 13-0879-T500). Flow cytometry data collection was performed on the Beckman Coulter Gallios Flow Cytometer (Beckman-Coulter). Flow cytometric data were analyzed using Kaluza Analysis Software (Beckman-Coulter).

### Cell blood counts

Mouse peripheral blood (50 μL) was collected into microtainer vials (Fisher, 02-669-33) via retroorbital bleeding. Blood samples were subsequently analyzed on the ABX SAS Pentra 60 (Horiba) cell blood counter to identify hematopoietic irregularities.

### Cell culture

MSCS cultures derived from mice were established by intrafemoral flushing with subsequent crushing of bone. Bone fractions were cultured in MesenCult Expansion Kit medium (Stemcell, 5513) in a T-75 flask, continuously refreshed, selected, and expanded for adherent cells to approximately 80% confluence. To isolate OSX+ MSCs, the GFP+ population was sorted using an Aria II Cell Sorter. GFP+ MSCs were subsequently returned to culture in Minimum Essential Medium α (Corning, catalogue number 15-012-CV) with 10% fetal bovine serum.

### RNA sequencing

MSCS cultures were plated in a six-well plate. After 48 hours, the cells were treated with 10 μM nutlin-3a for 24 hours. RNA was extracted by using the Direct-Zol Microprep kit (Zymo Research, R2060). RNA sequencing was performed as previously described ^43^.

### Fluorescence microscopy

Isolated tissues were fixed in 4% paraformaldehyde in PBS overnight. Next, bones were washed in PBS and decalcified in 14% EDTA solution for 10 days at 4°C. Bone samples were subsequently washed in PBS and immersed in 30% sucrose PBS solution overnight. Bones and soft tissues were transferred and submerged in optimal cutting temperature (OCT) compound (Tissue-Tek, 4583), after which the embedded tissue was cut into 5-μm sections with use of a cryostat. Slides were either stored as frozen slides at −80°C or further processed. Tissue slides were washed with PBS and then incubated in 3% bovine serum albumin for 1 hour at room temperature. After three subsequent 5-minute washes in PBS, primary antibody incubation against perilipin (Cell Signaling 9349S), leptin receptor (R&D Systems AF497), and p53 (CM5, Leica P53-CM5P-L) was performed at antibody-specific dilutions in a light-protected hydration chamber at 4°C. Secondary antibody staining against the primary antibody host species (Chicken anti-Goat IgG AF647, Invitrogen A-21469, and Donkey anti-Rabbit IgG AF647, Invitrogen A-31573) was performed at dilutions of 1:500 for 1 hour at room temperature. Tissue slides were washed thrice in PBS for 5 minutes, stained with 4′,6-diamidino-2-phenylindole (DAPI, Life technologies, D3571) at 1:750 for 5 minutes, and rinsed with PBS. Slides were mounted with VECTASHIELD Mounting Medium (Fisher, H-1000), and the cover slides were sealed with nail polish.

### Imaging and spectral deconvolution

Frozen sections and immunofluorescence-stained slides were imaged by using Vectra multispectral imaging system, version 2 (Akoya Biosciences). All samples were scanned at 40× magnification and visualized with a Phenochart slide viewer (Akoya Biosciences).

### Tissue preparation and immunohistochemical analysis

Immunohistochemical analysis was performed at room temperature by using the VECTASTAIN Elite ABC HRP Kit (Vector Laboratories, Pk-6101). Formalin-fixed paraffin-embedded slides were deparaffinized in two changes of xylene, followed by two changes of 100% ethanol, and then once through 95%, 75%, and 50% ethanol for 5 minutes per change. Endogenous peroxidase activity was subsequently inhibited by immersion in 3% H_2_O_2_ solution in methanol for 10 minutes. After two 5-minute washes in Tris-buffered saline (TBS), slides were immersed in Coplin jars with antigen retrieval solution and placed in an IHC-Tek Epitope Retrieval Steamer (IHC World, IW-1102) for 10 minutes. After a cool-down period of 20 minutes at room temperature, the slides were blocked for 60 minutes with 10% serum in 0.1% TBST. Primary antibody incubation for p53 (CM5, Leica P53-CM5P-L) was done overnight in 2% FBS in 0.1% TBST in a light-protected hydration chamber at 4°C. Upon primary antibody incubation, in addition to two 5-minute washes in TBS, slides were incubated in biotinylated secondary antibody solution for 30 minutes at room temperature. Next, after two 5-minute washes in TBS, slides were incubated for 30 minutes in ABC solution. Two 5-minute washes in PBS followed, after which the slides were incubated in 3,3′-diaminobenzidine solution until the desired signal intensity was reached. The diaminobenzidine solution was washed off with deionized H_2_O, and slides were next counterstained with hematoxylin for 80 seconds. Incubation of slides in lithium carbonate solution for 60 seconds followed, after which the slides were immersed in deionized H_2_O for 10 minutes. Slides were next immersed for two changes, respectively, of 95% ethanol and 100% ethanol for 90 seconds per change. Next slides were immersed three times for 5 minutes in xylene. Finally, the slides were mounted with Richard-Allan Scientific Mounting Medium (Thermo Scientific, 4112).

### TUNEL staining

TUNEL staining of frozen tissue slides was performed using the Click-iT Plus TUNEL Assay for In Situ Apoptosis Detection kit (Thermo Fisher Scientific, C10619). Briefly, solutions outlined by the kit were prepared before starting the staining procedure. Frozen tissue sections were brought to room temperature in PBS for 15 min, and the tissue was encircled with a PAP pen. Tissues were fixed in 4% formaldehyde in PBS at 37° C, washed twice for 5 min in PBS, and subsequently incubated for 15 min with proteinase K solution at room temperature. Next, slides were washed in PBS for 5 min, fixed again in 4% formaldehyde in PBS for 5 min at 37° C, and rinsed in deionized H2O. Incubation of slides with 100 μL of compound A for 10 min at 37°C followed, after which the tissue was incubated with 50 μL of TdT reaction mixture for 60 min at 37° C. After a deionized H2O rinse, slides were washed in 0.2 μm filtered 3% bovine serum albumin 0.1% PBST for 5 min, rinsed once more in PBS, and incubated with 50 μL of reaction cocktail for 30 min at 37° C. Tissue slides were rinsed in PBS and stained with DAPI at 1:750 for 5 min. Slides were mounted as described above.

### Skeletal morphometry

Trabecular and cortical bone parameters were characterized with use of micro-computed tomography (μCT) imaging. Briefly, age-sorted mice with genotypes of interest were scanned *in vivo*. Scans were conducted with a 13-μm pixel size in a Bruker Sky Scan 1276 μCT scanner. Femur data were further analyzed by using the CT Analyzer (CTan) program to assess trabecular and cortical bone microarchitecture.

### Statistical analyses

The mean and standard deviation for the indicated number of samples were calculated using GraphPad Prism6 software. The Student’s *t* test was used for comparative analysis between two groups. Analysis of variance was used to compare multiple groups. pvalues ≤ 0.05 were considered statistically significant.

## Data Availability

The data and materials used and/or analyzed for the current study are available upon request from the corresponding author.
